# National survey of the association of depressive symptoms with the number of off duty and on-call, and sleep hours among physicians working in Japanese hospitals: a cross sectional study

**DOI:** 10.1186/1471-2458-10-127

**Published:** 2010-03-12

**Authors:** Koji Wada, Toru Yoshikawa, Takahisa Goto, Aizan Hirai, Eisuke Matsushima, Yoshifumi Nakashima, Rie Akaho, Michiko Kido, Takashi Hosaka

**Affiliations:** 1 The committee for Physicians' Health, the Japan Medical Association, Japan; 2 Department of Preventive Medicine and Public Health Kitasato University School of Medicine, Sagamihara, Japan; 3 Institute for Science of Labour, Kawasaki, Japan; 4 Department of Anesthesiology and Critical Care Medicine Yokohama City University Graduate School of Medicine, Yokohama, Japan; 5 Chiba prefectural Togane Hospital, Togane, Japan; 6 Section of Liaison Psychiatry and Palliative Medicine, Graduate School of Medical and Dental Sciences, Tokyo Medical and Dental University, Tokyo, Japan; 7 Department of psychiatry, Mitsui Memorial Hospital, Tokyo, Japan; 8 Department of Psychiatry, Tokyo Metropolitan Cancer and Infectious disease Center Komagome Hospital, Tokyo, Japan; 9 Departments of Obstetrics & Gynecology, the Japanese Red Cross Medical Center, Tokyo, Japan; 10 Tokai University School of Medicine, Tokyo Hospital, Tokyo, Japan

## Abstract

**Background:**

Physicians' mental health may be adversely affected by the number of days of work and time spent on-call, and improved by sleep and days-off. The aim of this study was to determine the associations of depressive symptoms with taking days of off duty, hours of sleep, and the number of days of on-call and overnight work among physicians working in Japanese hospitals.

**Methods:**

A cross-sectional study as a national survey was conducted by mail. The study population was 10,000 randomly selected physicians working in hospitals who were also members of the Japan Medical Association (response rate 40.5%). Self-reported anonymous questionnaire was sent to assess the number of days off-duty, overnight work, and on-calls, and the average number of sleep hours on days not working overnight in the previous one month. Depressive state was determined by the Japanese version of the Quick Inventory of Depressive Symptomatology. Logistic regression analysis was used to explore the associations between depressive symptoms and the studied variables.

**Results:**

Among the respondents, 8.3% of men and 10.5% of women were determined to be depressed. For both men and women, depressive state was associated with having no off-duty days and averaging less than 5 hours of sleep on days not doing overnight work. Depressive state was positively associated with being on-call more than 5 days per month for men, and more than 8 days per month for women, and was negatively associated with being off-duty more than 8 days per month for men.

**Conclusion:**

Some physicians need some support to maintain their mental health. Physicians who do not take enough days-off, who reduced sleep hours, and who have certain number of days on-calls may develop depressive symptoms.

## Background

Physicians' mental health is essential not only for physicians themselves but also for patients. Many mental health issues have been reported for physicians such as fatigue, depression, and burnout [1-3], which may be due to too much stress, less organizational support in hospitals, and certain traits of physicians, including being perfectionists and workaholics [4,5].

Depression among physicians has been often reported among medical residents [6,7], and may develop due to long working hours [8,9]. Thus, some countermeasures, such as limiting the number of hours of work among medical residents have been implemented in some countries [10]. However, some senior physicians also work long hours and take less holidays because they feel guilty for taking days off in light of the shortage of physicians [5].

In Japan, some senior physicians work long hours and suffer from lost motivation, prolonged fatigue, depression, and in worst cases, death due to overwork [11,12]. The United States, Canada, and the United Kingdom have already started physicians' health programs which provide mental health support by phone counselling, encouraging organizational support in hospitals, and raising awareness of physicians of the importance of maintaining their own health [13-15].

The Japan Medical Association launched a project team for physicians' health focusing on physicians working in hospitals in 2008. The reason that the committee focused on physicians working in hospitals is that some hospitals face a shortage of physicians because some physicians have left hospitals to open clinics or to go to other hospitals with better working conditions, resulting in the closing of some departments or reducing the number of in-house beds, especially in rural areas [16]. In Japan most doctors work either hospitals or clinics as full time physicians. There is an urgent need to ensure an adequate number of physicians in hospitals. To build a framework for the physician health program in Japan, few studies have focused on mental health and working hours for Japanese physicians [1,3]. Thus, this study was performed as a national survey to identify the issues in physicians' health and work in Japan.

Working hours are difficult to measure since physicians often take time not only for caring patients, but also for studying or doing research. We focused on factors that could reflect real situations, such as the number of days off, number of days spent in overnight work and on-call, and the average number of sleep hours during not doing overnight work. These factors affect mental health for physicians, and could provide targets for interventions by hospitals and physicians themselves [1,17]. The aim of this study was to determine the associations of depressive symptoms with taking days of off duty, hours of sleep, and the number of days on-call and overnight work among physicians working in Japanese hospitals.

## Methods

We sent an anonymous, self-administered questionnaire by mail to 10,000 randomly selected physicians working in hospitals who were also members of the Japan Medical Association (75,951 physicians).

Questions included the number of days spent off-duty per month (none/1-4 days/5-7 days/8 days or more), the number of days spent on-call per month (none/1-4 days/5-7 days/8 days or more), the number of days of overnight work per month (none/once/2 to 3 times/4 to 5 times/6 times or more), and the average number of hours spent sleep when not doing overnight work (less than 5 hours/5 to less than 6 hours/6 to less than 7 hours/more than 7 hours) in the previous one month.

Physicians who were depressed were identified as those having a score of more than 11 on the Japanese version of the Quick Inventory Depressive Scale-Self Reported (QIDS-SR) [18,19] The QIDS-SR comprised 16 questions on a 4 point Likert scale and measured the severity of depressive symptoms for the previous 7 days. The range of scores of the QIDS-SR is from 0 to 27, and a score of 0 to 5 indicates no depressive symptoms, 6 to 10 indicates mild symptoms, 11 to 15 moderate, 16 to 20 severe, and 21 to 27 very severe depressive symptoms.

Logistic regression analysis was used to explore the association between depressive symptoms and the studied variables. We first examined the variables mentioned above by univariate analysis, and then adjusted for age and the study variables. All analyses were performed using the Statistical Package for the Social Sciences (SPSS) for Windows, ver. 15.0 [20].

### Ethics

The Human Research Committee at the Institute for Science of Labour gave approval the study protocol prior to it being conducted. Since the questionnaire was in an anonymous form, we assumed that participants agreed to participate in our study by returning their questionnaire.

## Results

Thirty one questionnaires did not reach the respondents and were returned. A total of 3,862 persons completed the questionnaire, and 176 replied but did not answer the questionnaire because they were not suited for this study. Among persons who did not complete the questionnaire, 62 persons (35.2%) provided reasons, of which 34 answered that they longer worked in hospitals, they were sick, or they were on maternity leaves. Fifty-six persons out of 176 who did not complete were over 70 years old. Thus, the adjusted response rate was 40.5%.

There were 3,025 men and 837 women who completed the questionnaire. Table 1 shows the characteristics of participants. The majority of men were 40 to 59 years old, and the majority of women were 30 to 49 years old. There were about 8 to 10 percent of respondents who did not have any days off in the previous one month, and about 20 percent of respondents had 8 or more days on-call in the previous one month.

**Table 1:** Characteristics of the participants by sex

	Men		Women	
	(n = 3,025)	(%)	(n = 837)	(%)
Age (years)				
24-29	38	(1.3)	51	(6.1)
30-39	417	(13.7)	300	(35.8)
40-49	876	(29.0)	270	(32.3)
50-59	967	(32.0)	141	(16.8)
60-69	464	(15.3)	55	(6.6)
70 or more	263	(8.7)	20	(2.4)
				
The number of days of off-duty per month				
None	299	(9.9)	70	(8.4)
1-4 days	1203	(39.8)	279	(33.3)
5-7 days	986	(32.6)	222	(26.5)
8 days or more	537	(17.7)	266	(31.8)
				
The number of days of on-call per month				
None	1338	(44.2)	476	(56.9)
1-4 days	669	(22.1)	149	(17.8)
5-7 days	379	(12.5)	59	(7.0)
8 days or more	639	(21.1)	153	(18.3)
				
Average sleeping hours for days not working overnight				
Less than 5 hours	263	(8.7)	83	(9.9)
5 to less than 6 hours	977	(32.3)	290	(34.6)
6 to less than 7 hours	1390	(46.0)	366	(43.7)
7 hours or more	395	(13.0)	98	(11.8)
				
The number of days of overnight work per month				
None	1261	(41.7)	480	(57.3)
Once	288	(9.5)	59	(7.0)
2 to 3 times	598	(19.8)	136	(16.2)
4 to 5 times	529	(17.5)	97	(11.6)
6 times or more	349	(11.5)	65	(7.8)
				
Score of the quick inventory depressive scale				
0-5	2054	(67.9)	550	(65.7)
6-10	719	(23.8)	199	(23.8)
11-15	199	(6.5)	66	(7.9)
16-20	41	(1.4)	18	(2.2)
21-27	12	(0.4)	4	(0.5)

Table 2 shows the associations between depressive symptoms and the studied variables. Among the respondents, 252 (8.3%) men and 88 (10.5%) women were determined to be in a depressive state with a score of 11 or more on the QIDS. There were more physicians in a depressive state among those without any days off-duty, those on-call for 8 days or more, and those with overnight work for 6 days of more in the previous one month.

**Table 2:** Associations between depressive symptoms and the studied variables in the previous one month

	Men				Women			
		
	Without depressive symptoms		With depressive symptoms		Without depressive symptoms		With depressive symptoms	
				
	n = 2773	(%)	n = 252	(%)	n = 749	(%)	n = 88	(%)
The number of days of off-duty per month								
None	256	(85.6)	43	(14.4)	54	(77.1)	16	(22.9)
1-4 days	1088	(90.4)	115	(9.6)	253	(90.7)	26	(9.3)
5-7 days	915	(92.8)	71	(7.2)	201	(90.5)	21	(9.5)
8 days or more	514	(95.7)	23	(4.3)	241	(90.6)	25	(9.4)
								
The number of days of on-call per month								
None	1260	(94.2)	78	(5.8)	445	(93.5)	31	(6.5)
1-4 days	615	(91.9)	54	(8.1)	130	(87.2)	19	(12.8)
5-7 days	338	(89.2)	41	(10.8)	50	(84.7)	9	(15.3)
8 days or more	560	(87.6)	79	(12.4)	124	(81.0)	29	(19.0)
								
Average sleeping hours for days not working overnight								
Less than 5 hours	216	(82.1)	47	(17.9)	65	(78.3)	18	(21.7)
5 to less than 6 hours	892	(91.3)	85	(8.7)	256	(88.3)	34	(11.7)
6 to less than 7 hours	1300	(93.5)	90	(6.5)	338	(92.3)	28	(7.7)
7 hours or more	365	(92.4)	30	(7.6)	90	(91.8)	8	(8.2)
								
The number of days of overnight work per month								
None	1178	(93.4)	83	(6.6)	440	(91.7)	40	(8.3)
Once	264	(91.7)	24	(8.3)	53	(89.8)	6	(10.2)
2 to 3 times	540	(90.3)	58	(9.7)	115	(84.6)	21	(15.4)
4 to 5 times	482	(91.1)	47	(8.9)	87	(89.7)	10	(10.3)
6 times or more	309	(88.5)	40	(11.5)	54	(83.1)	11	(16.9)

Table 3 shows the univariate and multiple logistic regression analyses regarding factors on depressive symptoms. In the univarite analysis, having no days off-duty per month, being on-call 8 times or more per month, averaging less than 5 hours of sleep per night not doing overnight work, and working overnight 6 or more times per month were associated with depressive state in both men and women. For men, being off-duty 8 days or more per month was negatively associated with depressive state, the number of days of overnight work for 2 to 3 times, and being on-call 5 to 7 days per month was positively associated with depressive state.

**Table 3:** Logistic regression analysis of regarding factors in the previous one month on depressive symptoms

	Men				Women			
		
Variables	Crude		Adjusted		Crude		Adjusted	
	OR	(95% CI)	OR^a^	(95% CI)	OR	(95% CI)	OR^a^	(95% CI)
The number of days of off-duty per month								
None	2.13	(1.42 to 3.19)	1.62	(1.05 to 2.52)	3.30	(1.64 to 6.64)	2.39	(1.10 to 5.19)
1-4 days	1.30	(0.96 to 1.75)	1.10	(0.80 to 1.51)	1.02	(0.57 to 1.83)	0.75	(0.40 to 1.42)
5-7 days	1.00		1.00		1.00		1.00	
8 days or more	0.52	(0.32 to 0.86)	0.53	(0.31 to 0.90)	0.81	(0.44 to 1.51)	1.08	(0.55 to 2.12)
								
The number of days of on-call per month								
None	1.00		1.00		1.00		1.00	
1-4 days	1.36	(0.95 to 1.95)	1.27	(0.88 to 1.84)	1.53	(0.84 to 2.79)	1.55	(0.80 to 3.00)
5-7 days	1.91	(1.28 to 2.84)	1.75	(1.15 to 2.64)	1.41	(0.57 to 3.49)	1.14	(0.43 to 3.06)
8 days or more	2.19	(1.58 to 3.04)	1.77	(1.24 to 2.52)	2.41	(1.41 to 4.1)	1.80	(0.98 to 3.28)
								
Average sleeping hours for days not working overnight								
Less than 5 hours	2.79	(1.96 to 3.95)	2.70	(1.82 to 4.03)	2.65	(1.47 to 4.78)	2.38	(1.11 to 5.10)
5 to less than 6 hours	1.33	(0.98 to 1.81)	1.20	(0.88 to 1.65)	1.62	(0.96 to 2.74)	1.54	(0.89 to 2.67)
6 to less than 7 hours	1.00		1.00		1.00		1.00	
7 hours or more	1.10	(0.71 to 1.70)	1.32	(0.84 to 2.08)	1.12	(0.49 to 2.54)	1.08	(0.46 to 2.49)
								
The number of days of overnight work per month								
None	1.00		1.00		1.00		1.00	
Once	1.21	(0.75 to 1.95)	1.00	(0.60 to 1.65)	1.06	(0.40 to 2.79)	0.84	(0.29 to 2.40)
2 to 3 times	1.47	(1.04 to 2.08)	1.14	(0.74 to 1.67)	2.06	(1.17 to 3.62)	1.65	(0.86 to 3.17)
4 to 5 times	1.33	(0.92 to 1.94)	1.11	(0.74 to 1.68)	1.31	(0.63 to 2.71)	1.21	(0.53 to 2.77)
6 times or more	1.77	(1.19 to 2.64)	1.33	(0.86 to 2.07)	2.37	(1.15 to 4.89)	1.90	(0.82 to 4.42)

OR, odds ratio; CI, confidence interval

^a^ORs are adjusted for age and all other variables in the table

For both men and women, depressive state was significantly associated with no days off-duty per month (odds ratio 1.62, 95% confidence interval 1.05 to 2.52 for men; 2.39, 1.10 to 5.19 for women), and sleep an average of less than 5 hours per night for days not doing overnight work (2.70, 1.82 to 4.03 for men and 2.38, 1.11 to 5.10 for women). For men, depressive state was associated with being on-call for 5 to 7 days per month (1.75, 1.15 to 2.64), and 8 days or more per month (1.77, 1.24 to 2.52), and being off-duty 8 days or more per month (0.53, 0.31 to 0.90). For women, depressive state was weakly associated with being on-call for 8 days or more per month (1.80, 0.98 to 3.28).

## Discussion

In this study, 8.3% of men and 10.5% of women were identified as being in depressive state. Eight to 9% of respondents did not have any holidays in a month, and about 20% of the respondents did on-call 8 days or more per month. We also report the associations of depressive state with lack of days-off and with being on-call more than 5 times per month for male and more than 8 times per month for female physicians working in Japanese hospitals. Eight to 9% of respondents had slept less than 5 hours per night during days not doing overnight work which was also associated with a depressive state.

The study population were selected from 75,951 physicians working in hospitals and who were also members of the Japanese Medical Association. According to the statistics of the Ministry of Health, Labour, and Welfare, Japan there were 168,327 physicians working hospitals out of the all the 277,927 physicians in 2006 [21]. We do not have any information about the physicians who are working in hospitals but not being a member of the Japan Medical Association. However, the generalizability of this study could be ensured comparing with other small scale studies.

Depressive symptoms among physicians may not only affect their own health, but may also threaten patients' safety. Physicians often consider it a stigma against consulting to other physicians about their own health conditions [5]. It often takes time to seek help from other physicians and in the worst case, failure could result in committing suicide [22]. With regard to patient safety, there have been some studies showing that burnout and fatigue, which also could represent a symptom of depression, were associated [23,24]. Adler et al. indicated depression could affect job performance [25]. Since a number of physicians in Japan were found to have depressive symptoms, it will necessary to provide organizational services to support such physicians for their health and patients' safety.

Some physicians tend to feel guilty if they take days-off [5]. Some physicians in Japan are likely to visit hospitals to take care of their patients even on their holidays. Taking days off could prevent fatigue and is essential for maintaining a work life balance [26]. About 40 to 50% of physicians had fewer than 4 days off per month. In our analysis we chose 5 to 7 days off as the reference because we would like to encourage physicians to take more days off. This was prompted by the fact that about 50% of our respondents took fewer than 4 days off per month. We also stressed that 8 or more days off per month, which are ideal, could be a factor preventing depressive symptoms. Arrangements need to be set in place so that physicians can take more days off.

"On-call" physicians are contacted by phone as needed. Senior physicians or specialists such as neurosurgeons, cardiologists, and orthopedic surgeons provide services to medically screen and stabilize emergency conditions. Usually, physicians on-call should have their mobile phones on hand at any time even though they do not need to stay in the hospital. Active on-call hours affect the well-being of physicians [17]. In this study, there was an association between depressive symptoms and being on-call 5 days or more per month for men, and 8 days or more per month for women. About 20 to 30% of physicians were on-call 5 days or more per month. In this study, gynecologists (47.8%), urologists (32.4%), neurosurgeons (42.9%), and anesthesiologists (35.1%) were on-call for 8 days or more per month. Hospitals should consider reducing the number of days on-call.

Surprisingly, the number of days of overnight work was not associated with depressive symptoms. This result does not indicate that overnight work does not harm mental health for physicians. There have also been some reports that showed an association between overnight work and medical safety and accidents [27,28]. In the univarite analysis, overnight work more than 6 times per month was associated with depressive symptoms for both men and women. In Japan, each hospital often has a limited numbers of specialists which results in an increase in the number of days of overnight work per physician depending on the number of staff. Centralization of health care provisions for some specializations such as obstetrics and pediatrics has just begun in order to reduce the workload in Japan [29].

Lack of sleep hours was associated with depressive symptoms and fatigue [30-32]. Limiting the number of hours of work among medical residents has been reported to be ineffective for either improving conditions such as patient safety or preventing fatigue [33]. This could be because medical residents do not sleep enough even though their working hours are limited. Thus, education on the importance of sleep for physicians is also needed. About 10 percent of respondents slept less than 5 hours per month on days not doing overnight work. They need to recognize the importance of sleep.

The present study has some limitations. First, since this was a cross-sectional study, we could not confirm causal relationships. Second, there were some other important factors which should be adjusted such as their specialties. Third, average sleeping hours were less than 5 hours for days that were not worked overnight; this could have been reflected in depressive symptoms.

## Conclusion

We identified that some physicians need some support to maintain mental health. Physicians who do not take enough days-off, who have reduced sleep hours, and who have an increased number of days on-calls may develop depressive symptoms among Japanese physicians working in hospitals.

The present study highlighted an issue to be addressed for physicians' health programs in Japan. Hospitals should take steps to ensure that physicians can take more days off work, get adequate sleep, and reduce days of on-call service. Physicians themselves should also recognize the importance of these factors to maintain their own health and provide sustainable health care.

## Competing interests

The authors declare that they have no competing interests.

## Authors' contributions

KW, TY, and TH planned the study and drafted the manuscript, EM, YN, and RA contributed to the part of depressive symptoms and TG, AH, and MK contributed to the part of working conditions in hospitals. KW performed the statistical analysis. All the authors read and approved the final manuscript.

## Pre-publication history

The pre-publication history for this paper can be accessed here:

http://www.biomedcentral.com/1471-2458/10/127/prepub
